# Disseminated peritoneal leiomyomatosis: a rare cause of enigmatic peritoneal masses

**DOI:** 10.1259/bjrcr.20150252

**Published:** 2016-07-28

**Authors:** Shivaprakash Basavanthaiah Hiremath, Geena Benjamin, Amol Anantrao Gautam, Sathibhai Panicker, Aji Rajan

**Affiliations:** ^1^Department of Imaging Sciences and Interventional Radiology, Sree Chitra Tirunal Institute for Medical Sciences and Technology, Trivandrum, India; ^2^Department of Radiodiagnosis, Pushpagiri Institute of Medical Sciences and Research Centre, Tiruvalla, India; ^3^Department of Pathology, Pushpagiri Institute of Medical Sciences and Research Centre, Tiruvalla, India

## Abstract

Disseminated peritoneal leiomyomatosis (DPL) is an unusual extrauterine form of leiomyoma that has been found to coexist with its intrauterine counterpart in individuals who have previously undergone laparoscopic myomectomy. The presence of extrauterine masses with the density of smooth muscle on CT imaging and/or with a low signal intensity similar to that of smooth muscle on *T*_2_ weighted MRI in a patient presenting with associated intrauterine leiomyoma and/or a history of previous laparoscopic myomectomy suggests the possibility of DPL. Imaging studies help in diagnosing and delineating the location and extent of the lesion and also follow-up the masses to look for sarcomatous transformation. Here we report the case of a 43-year-old female who presented initially with right lower quadrant pain. Her CT scan and MRI demonstrated a fundal fibroid with multiple intraperitoneal soft tissue masses of similar appearance and contrast enhancement in the sigmoid mesocolon, the left paracolic gutter and adjacent to the ascending colon. The suspected diagnosis of DPL was confirmed during abdominal hysterectomy, bilateral salphingo-oophorectomy and excision of peritoneal masses.

## Clinical presentation

A 43-year-old female with a history of ulcerative colitis presented with lower abdominal pain of 3-day duration. The pain was continuous, dull aching in nature and associated with low grade fever. The patient also complained of vaginal discharge but with no history of loose stools or dysuria. She gave a history of undergoing laparoscopic myomectomy twice in the past.

All laboratory investigations were within normal limits. Clinical examination revealed a firm mass of 16-week size in the hypogastrium extending into the left iliac fossa with restricted mobility. Per vaginal examination showed the uterus to be of 16-week size with restricted mobility. A firm mass, separate from the uterus was felt through bilateral fornices and the posterior fornix.

## Investigations

**Figure 1.**
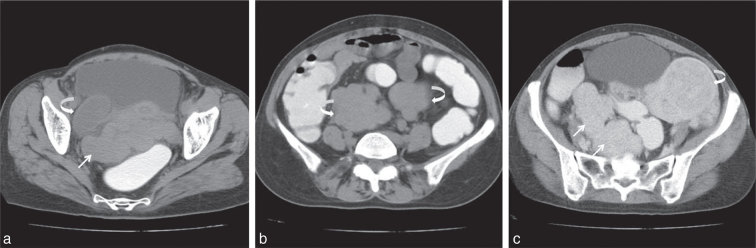
(a) Axial plain CT section with rectal contrast shows lobulated soft tissue attenuation lesion posterior to the uterus (arrow) with a fluid attenuation lesion in the right adnexa (curved arrow). (b) Axial plain CT section with rectal contrast shows lobulated soft tissue attenuation lesions in the sigmoid mesocolon and adjacent to the ascending colon (curved arrows). (c) Axial post-contrast CT section shows lobulated, enhancing soft tissue attenuation lesion in the uterus (curved arrow) with a similar enhancing lesion abutting the sigmoid colon (arrows).

Abdominal CT scan showed a fundal fibroid involving the anterior wall with a large multilobulated soft tissue density mass posterior to the uterus (Figure 1a). Similar small mass lesions were noted involving the sigmoid mesocolon, left paracolic gutter and adjacent to the ascending colon (Figure 1b). Post-contrast imaging showed homogeneous enhancement of the lesions similar to that of the fundal fibroid (Figure 1c). No evidence of bowel wall thickening, mesenteric hypervascularity or fat proliferation was noted.

**Figure 2.**
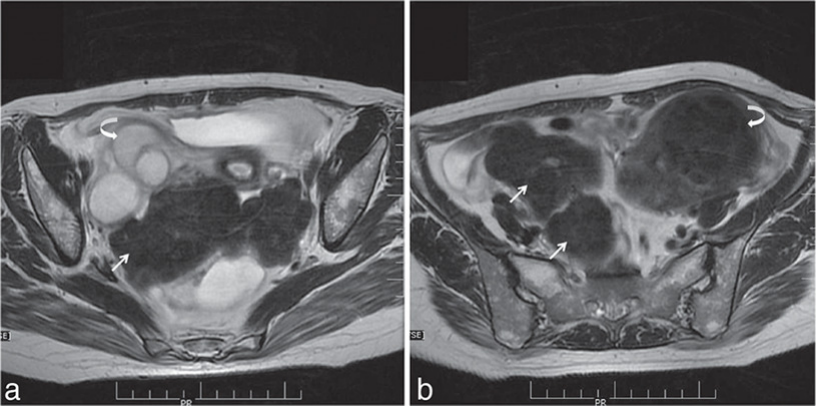
(a) Axial *T*_2_ respiratory triggering (RTr) image of the pelvis shows a lobulated hypointense lesion posterior to the uterus (arrow) with a hyperintense lesion in the right adnexa (curved arrow). (b) Axial *T*_2_ RTr image of the pelvis shows a lobulated hypointense lesion adjacent to the caecum (arrows) with a lesion of similar intensity in the uterus (curved arrow).

MRI showed multiple intramural fibroids exhibiting *T*_1_ and *T*_2_ weighted hypointensities (Figure 2a). Similar signal intensity extrauterine lesions were noted in the rectouterine pouch, sigmoid mesocolon and left paracolic gutter (Figure 2b). A right-sided hydrosalphinx was also seen (curved arrow in Figures 1a and 2b). There was no evidence of hydronephrosis. Cine loops of post-contrast CT and axial *T*_2_ respiratory triggering (RTr) images are available as Supplementary videos A and B.

**Figure 3.**
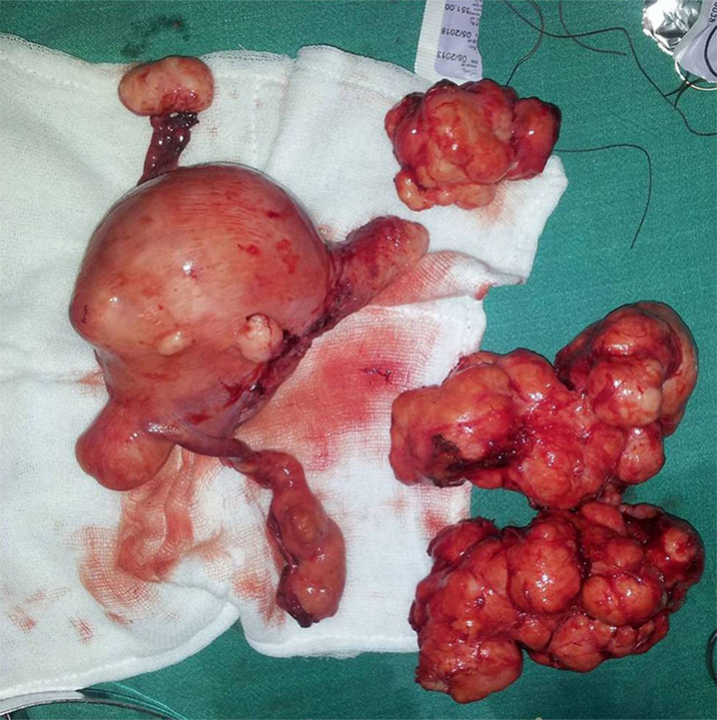
Post-operative specimen shows an enlarged uterus with bosselated surface. Multiple lobulated extrauterine lesions were also removed.

**Figure 4.**
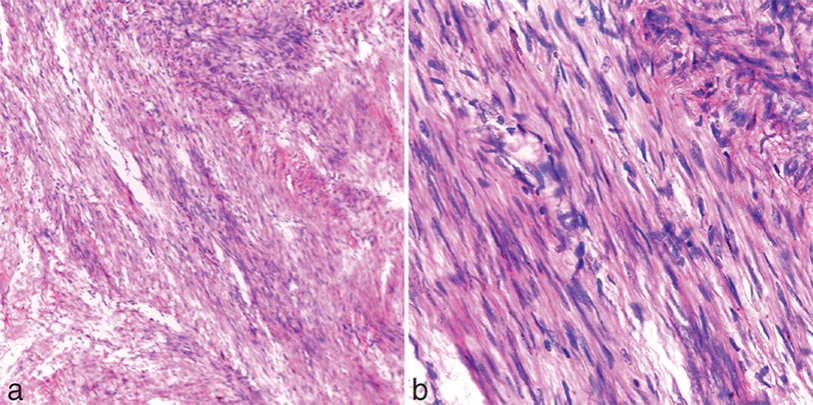
(a) Histopathological section from the intrauterine lesions shows a neoplasm composed of interlacing bundles and whorls of benign spindle cells with focal areas of hyalinization. (b) Histopathological section from an extrauterine lesion shows a whorled pattern of smooth muscle bundles separated from each other by vascularized connective tissue and no pleomorphism.

The imaging findings were suggestive of uterine leiomyoma with coexisting disseminated peritoneal leiomyomatosis. The patient underwent total abdominal hysterectomy, bilateral salphingo-oophorectomy and removal of peritoneal leiomyomata (Figure 3). Histological examination of the intrauterine lesions showed a tumour composed of interlacing bundles and whorls of benign spindle cells with focal areas of hyalinization (Figure 4a). Multiple extrauterine lesions showed a whorled pattern of smooth muscle bundles separated from each other by vascularized connective tissue and no pleomorphism (Figure 4b). Acute suppurative endosalphingitis producing pyosalphinx was noted involving the right fallopian tube.

## Differential diagnosis

The presence of characteristic *T*_2_ hypointensity of the extrauterine masses similar to that of smooth muscle, with associated intrauterine leiomyoma or previous laparoscopic myomectomy, is suggestive of disseminated peritoneal leiomyomatosis.

## Outcome and follow-up

There has been no recurrence of intra- or retroperitoneal masses post surgery.

## Discussion

Leiomyomas are benign smooth muscle tumours that arise from the overgrowth of smooth muscle and connective tissue, usually from the uterus, small bowel and the oesophagus. Uterine leiomyomas are the most common gynaecological tumours noted to occur in 20% to 30% of women beyond the age of 35 years.^1,2^ Uncommon growth patterns of leiomyoma include disseminated peritoneal leiomyomatosis (DPL), benign metastasizing leiomyoma, intravenous leiomyoma, retroperitoneal leiomyoma and parasitic leiomyoma.

## Incidence and aetiopathogenesis

DPL (also known as leiomyomatosis peritonalis disseminata) is an extremely rare benign entity characterized by the presence of multiple leiomyomas throughout the peritoneal cavity. The first case was reported by Willson and Peale^3^ in 1952; however, the entity was clearly described and named by Taubert et al^4^ in 1965. About 140 cases of DPL had been reported by 2010 in the literature.^5^

Although the aetiopathogenesis is controversial, strong hormonal association is attributed owing to coexisting factors such as pregnancy, long-term use of oral contraceptives and oestrogen-producing tumours.^6^ The basic pathogenesis of DPL is thought to be a multicentric metaplastic change involving the connective tissue of the peritoneum in the abdominal cavity owing to an abnormal response to hormonal stimulation.^7^ This hypothesis can account for the association of DPL with endometriosis, as the subcoelomic mesenchyme is capable of differentiating into various tissues, including endometrial glandular epithelium. Laparoscopic removal of uterine leiomyomas has also been implicated in the development of DPL owing to spread of tumour cells along the surgical tract.^8^

## Clinical and imaging features

Most of the patients with DPL are asymptomatic, with lesions detected incidentally during surgery (caesarean section, laparotomy or laparoscopy). Others usually present with non-specific symptoms such as abnormal, heavy uterine bleeding and lower abdominal pain or discomfort. Less common presentations include increased frequency of micturition, mass per abdomen and symptoms of obstructive uropathy.

Imaging studies delineate the presence of intrauterine leiomyoma as well as the extent and location of peritoneal deposits. Multiple small subcentimetre peritoneal nodules are below the resolution of imaging techniques and incidental detection is not rare. Ultrasonography and CT scan in patients with DPL show multiple, solid and complex soft tissue masses that are usually large and similar in morphology to uterine leiomyoma. These lesions show heterogeneous attenuation with variable enhancement as that of uterine leiomyoma on post-contrast studies.

MRI features include multiple masses with hypointense signal similar to that of skeletal and smooth muscles on *T*_1_ and *T*_2_ weighted images, which show variable post-contrast enhancement. Follow-up imaging and surveillance is mandatory as sarcomatous transformation has been reported in a few cases.^9^ Positron emission tomography shows absence of fludeoxyglucose (FDG) uptake in nodules of DPL. Positron emission tomography is therefore a problem-solving tool in differentiating DPL from malignant peritoneal disease, which classically shows avid FDG uptake.^10^

## Treatment and prognosis

The mainstay of treatment involves curbing the hormonal influence on DPL, which includes a conservative approach and gonadotropin-releasing hormone agonist or surgical castration with or without removal of leiomyomatous masses.^6^ The conservative approach is considered for its benign clinical course when aggressive surgery is not feasible, as in younger age or for want of children. In view of the reported cases of recurrence and sarcomatous transformation, close surveillance of these patients is mandatory.

## Other uncommon subtypes of leiomyoma

Benign metastasizing leiomyoma presents as multiple well-differentiated lesions at sites farther than the uterus. Lesions most commonly affect the lungs. Other rare sites include heart, brain, lymph nodes and skin.^11^ Pathological hypothesis includes haematogenous dissemination of uterine leiomyoma, with an alternate theory proposing multifocal synchronous metaplasia.^12^ Imaging features include pulmonary nodules that can be solitary to multiple lung masses that mimic metastasis from a malignant tumour. Intralesional cavitation and calcification are rare, with occasional presentation as pneumothorax. Presence of uterine leiomyoma or history of hysterectomy with previous diagnosis of leiomyoma supports the diagnosis. However, definite evidence is based on image-guided core biopsy.

Intravenous leiomyomatosis is rare, benign smooth muscle proliferation within the uterine or systemic veins beyond the confines of leiomyoma. Tumoral growth is usually into the uterine and parametrial veins, with rare extension into systemic veins and cardia.^13^ The lesion appears as a filling defect *i.e.* thrombus in the great veins, inferior vena cava and sometimes extending into the right atrium, which is demonstrated by echocardiography. It shows hypointense signal on *T*_1_ and *T*_2_ weighted MRI, with enhancement on post-contrast images. Differential diagnoses include bland thrombus, tumoral thrombus with visualized primary malignancy and leiomyosarcoma.^14^

Multiple pelvic retroperitoneal lesions with concurrent uterine leiomyoma are suggestive of retroperitoneal leiomyomatosis. Rarely, leiomyomas adhere to adjacent structures (broad ligament or omentum) to derive vascular supply, hence loosing attachment to the uterus, and are termed parasitic leiomyomas.^15^ Ultrasound helps demonstrate concurrent uterine lesions and non-ovarian origin of extrauterine leiomyomas and aids guided biopsy for histological confirmation before surgery.

## Learning points

1. DPL is a rare benign entity characterized by multiple leiomyomas in the peritoneal cavity seen usually in the presence of uterine leiomyoma or history of hysterectomy due to previous leiomyoma.
2. Characteristic *T*_1_ and *T*_2_ hypointense signals similar to smooth muscle in uterine leiomyoma with variable post-contrast enhancement is the diagnostic finding that helps rule out differential possibilities of infectious and malignant aetiologies.
3. Positron emission tomography is a key modality to differentiate DPL from malignant peritoneal disease, which demonstrates the absence of uptake in nodules in DPL and avid FDG uptake in malignancy.
4. Close surveillance of patients is mandatory in view of the reported cases of recurrence and sarcomatous transformation.

## Consent

Informed consent has been obtained from the patient after explaining about the publication and usage of images.
